# Reports on the Hospitals of the United Kingdom

**Published:** 1910-01-01

**Authors:** Henry Burdett


### REPORTS
### ON THE
### Hospitals of the United Kingdom.

BY SIR HENRY BURDETT, K.C.B., K.C.V.O.

### XVII.—BROWNLOW HILL POOR LAW INFIRMARY, LIVERPOOL.

THIS famous Poor Law Infirmary, with its glorious memories of Agnes Jones and other pioneers, whose example and work have made the modern Poor Law hospital of to-day possible, presents a scandalous contrast to the up-to-dateness of the Royal Infirmary, Liverpool, supported by voluntary contributions. We suppose that there are Socialists in Liverpool who are fully persuaded that rate or State supported hospitals would be a blessing to the community. If there be, we invite them to pay a visit to Brownlow Hill to see what rate support and rate control mean as exhibited in the maternity department of this Poor Law Infirmary. There they will see girls of sixteen, and young women who are physically healthy, crowded together in the same wards with older women who may be suffering from contagious diseases of the most repulsive kind. How it has come to pass that such things should be permitted in a city like Liverpool, which has a deservedly high reputation in the outside world for the humanity of its chief citizens, is a question for each member of the Board of Guardians to answer. We are bound to say that we did not believe that such things are possible, even under the Poor Law in the worst administered and most callously conducted institution managed by a Board of Guardians. To find it going on without remedy or remorse in the Poor Law Infirmary where Agnes Jones laboured, and where a number of devoted women have striven with much self-denial to follow in her footsteps, is to bring vividly to the mind of every thinking man and woman in Liverpool and throughout the country what the poor may be subjected to in rate-supported hospitals.

### INSIDE THE WARDS.

It is a remarkable fact that classification of the inmates takes place in regard to all except those in the maternity wards. We may point out that although classification of this kind is essential for moral reasons in the general wards of a Poor Law Infirmary, in the maternity wards it is doubly necessary on moral and hygienic grounds; no Board of Guardians has the right to subject any inmate of a Poor Law Infirmary in any department to the risks of contracting a loathsome contagious disease, much less to permit infants to be subjected to such risks. We could wish that some inmate would test in a court of law the liability for damages of individual members of such boards as have subjected innocent poor people to the risks referred to. It is true that in regard to operations Brownlow Hill is better off than the Leeds Poor Law Infirmary, mainly owing to the zeal and energy of the visiting surgeon, Mr. Alexander, who has shown commendable ingenuity in creating a temporary theatre out of the garret or open-roofed space he has secured for the purpose at the top of one of the ward pavilions. The wards in these pavilions, with the exception of the old fever wards which are plastered, have walls of rough, unplastered bricks. These have been painted in two colours, with relatively good effect; but the hygienic condition of every one of these wards is unsatisfactory; dust rapidly accumulates and has constantly to be brushed off, so that the atmosphere of the wards is necessarily often charged with particles which, to say the least, cannot be healthy or good for the inmates. The ward floors are made of pitch pine; they display signs of wear, as well they may, for they have been down for so many years that it is impossible to say their exact age. It is certain that there must be dust-filth under these floors containing organisms representing most of the diseases known to hospitals. The equipment of the wards is quite out of date. The beds are made of fibre, which loses its elasticity after three months' wear, so the majority of the beds are hard, whilst the only pillows are of flock, which must add to the sufferings of many poor patients who are seriously ill. Flock is a material which ought never to be used in a hospital, and we could wish that every member of the Brownlow Hill Board of Guardians might be compelled to have none but fibre mattresses and flock pillows upon his own bedstead at home for the rest of his natural life.

### DISGRACEFUL LAVATORIES AND BATHROOMS.

The wards are, however, presentable places compared with the adjoining lavatories and bathrooms, most of which are so bad as to be, we believe, without parallel in any other infirmary. We imagine that the people of Liverpool will hardly credit that these places are for the most part dark and evil smelling. One bathroom which, we understood, is typical of several, is a dark, dirty-looking interior space, containing two baths, and dependent for light and air upon openings and glass in a partition which divides it off from the urinals and closets, whereby it alone communicates directly with the outer air. In another instance urinal and w.c.'s were shockingly bad; a number of men were smoking there when we entered them. These defects are notorious, and of course are due in a measure to the fact that the buildings of this Poor Law Infirmary at Brownlow Hill, or most of them, are so out of date and hygienically improper for the treatment of disease, that they should long ago have been replaced by modern, up-to-date wards worthy of the city of Liverpool. The mischief of it is that apart from the impropriety of treating patients, however poor, in such buildings, the moral effects upon a patient compelled to live under the conditions we have described must be grievous indeed. Is all spirit of citizenship, all sense of the responsibility attaching to the office of a guardian, all knowledge or conscience, all power of apprehension of the evils and cruelty which the existing state of affairs in the Brownlow Hill Poor Law Infirmary reveals, lost by every man and woman who belongs to the Brownlow Hill Board? Whether this be so or not, a very heavy responsibility rests upon the ratepayers who allow poor patients to be retained under the conditions described. It is not credible that the ratepayers, and possibly the members of the Board of Guardians itself, have realised the actual condition of the Brownlow Hill wards and their adjuncts, for otherwise it must have been impossible that such buildings should have been allowed to be occupied for so long a period by sick people. seeing that they stand within a stone's throw of the Liverpool Royal Infirmary.

### The Nursing Staff.

Economists are apt to contend that all palliatives for existing evils are false economy, and our inspection forced us to realise that the very excellence of the work done here by the nursing staff may have actually retarded the scrapping of these buildings. Had it not been for the zealous and self-denying labours of the noble women who have worked under such grave discouragements and difficulties for so many years, these buildings must, indeed, have proved hygienically impossible. It is a bright feature of this Poor Law infirmary that the nursing staff should be well housed; that it should have an efficient nursing home; that the nurses' meals should be supplied from a separate kitchen under the control of the lady superintendent, and that care has been taken to provide everything possible to promote the comfort and the health of the nurses. The supervision of the kitchen and supplies, and the general organisation throughout the home reflect the greatest credit upon the lady superintendent, Miss Stuart, and her staff. It is just, too, to give the Guardians due credit for having voted the necessary funds and accepted the recommendation of their officers in regard to these matters. We have no doubt that a nurse trained at Brownlow Hill, who completes her training and gets her certificate, must necessarily be most efficient, should she elect to enter the field of private nursing and devote her life to this branch of her profession, There is nothing like hard work under great difficulties to build up character and enable the probationer to develop into a woman of practical resource, free from fads and an excessive opinion of the importance of her own personal comfort and requirements. Hence it is that a nurse who has received her training in the school of a well-organised Poor Law infirmary is very often most popular and efficient. We have no doubt that Brownlow Hill has turned out a large number of excellent nurses, whose services have proved of the utmost value not only to Liverpool but to patients in private families in many parts of the country.

### Why Death has not Swept these Wards.

Will the reader please try to realise the strain under which the Lady Superintendents and Nurses must have worked for many years in the Brownlow Hill Infirmary? Had it not been for their devotion and presence it is certain that an epidemic of disease would have swept the wards, and Death would have reaped a multitude of lives. Everywhere there is evidence that, so far as administration and devotion to duty on the part of the nursing staff and their superiors is concerned, everything is done that can make things as good as possible for the patients, and done cheerfully and well. What amazes us is to find that the responsible authorities, who, it is presumed, must occasionally visit the wards, lavatories, baths, and so forth of this Poor Law infirmary, should have failed to realise the condition of these buildings, or, realising, should have failed to do their duty by condemning the buildings long ago. An inquiry by the Local Government Board is, we understand, about to be held into the present condition of the Brownlow Hill Workhouse, and we hope that inquiry will extend to the Poor Law infirmary buildings also. The prompt replacement of the present buildings by a modern, up-to-date infirmary is a matter so pressing that it would be an advantage to have it taken in hand even without the stimulus of a Government inquiry.

**The Gloucester General Infirmary** will be reported on next week.

### THE NATION'S TEETH.

The first Australian Dental Congress was held in Sydney nearly three years ago. The second sat in Melbourne this year from October 25 to 29. Over 300 dentists, with a sprinkle of women among them, attended from the six Australian States and New Zealand. The members of the Congress were entertained at luncheon by the Lord Mayor of Melbourne. The first day and in the afternoon the proceedings were opened by his Excellency the Governor-General at the University. Among the speakers were the Prime Minister, Mr. Alfred Deakin, and Sir John Madden, Chief Justice and Lieutenant-Governor, who congratulated the dentists upon their sturdy independence in having provided a splendid college and hospital out of their own pockets. Demonstrations were given at the Dental College every morning and papers read and discussed in the afternoons, Dr. Springthorpe, Dean of the Faculty of Dentistry and President of the Dental Board—as also of the Association of Trained Nurses—presiding at most of the sittings. Gaiety of some kind was provided each evening for both visitors and hosts. The military authorities of the Commonwealth are now giving consideration to the teeth of the Army, and it is probable that a dental corps somewhat on the lines of the present military medical service will be formed.

